# Nanomedicine for Inner Ear Diseases: A Review of Recent* In Vivo* Studies

**DOI:** 10.1155/2017/3098230

**Published:** 2017-10-10

**Authors:** Dong-Kee Kim

**Affiliations:** Department of Otolaryngology-Head and Neck Surgery, Daejeon St. Mary's Hospital, College of Medicine, The Catholic University of Korea, Daejeon, Republic of Korea

## Abstract

Nanoparticles are promising therapeutic options for inner ear disease. In this report, we review* in vivo* animal studies in the otologic field using nanoparticles over the past 5 years. Many studies have used nanoparticles to deliver drugs, genes, and growth factors, and functional and morphological changes have been observed. The constituents of nanoparticles are also diversifying into various biocompatible materials, including poly(lactic-co-glycolic acid) (PLGA). The safe and effective delivery of drugs or genes in the inner ear will be a breakthrough for the treatment of inner ear diseases, including age-related hearing loss.

## 1. Introduction

Hearing loss is a common feature of many inner ear conditions, including presbycusis, sudden sensorineural hearing loss (SSNHL), genetic diseases, noise-induced hearing loss, ototoxic hearing loss, and autoimmune inner ear disease. The prevalence of hearing loss due to these inner ear diseases is increasing because of an increase in life expectancy, exposure to noise, and the use of medicines such as anticancer drugs.

These inner ear diseases remain intractable, and treatment results are poor. One of several reasons for their intractability is that the drug does not readily reach the inner ear. When a drug is administered systemically, it must cross the blood labyrinth barrier (BLB) to reach the inner ear [1]. However, only a small amount of the drug crosses the BLB and reaches the inner ear. Therefore, high doses of medication must be administered to achieve the appropriate drug concentration in the inner ear and have a therapeutic effect. However, systemic administration of high drug concentrations, particularly steroids, has numerous side effects.

Intratympanic drug injection has been used to address these problems and has become the standard treatment for Meniere's disease and sudden deafness [2, 3]. In some ways, the inner ear is well suited to local delivery of drugs. Local delivery bypasses the BLB, allowing drugs to reach their intended target at a lower dose. Thus, higher drug concentrations can be achieved while systemic effects are minimized. However, the drug concentration in the inner ear obtained through intratympanic drug injection remains low.

Several methods have been proposed to improve the efficiency of intratympanic drug injection, such as use of the Silverstein Microwick (Micromedics, St. Paul, MN), microcatheter implantation, hydrogels, and nanoparticles [4]. In this report, we focus on* in vivo* studies in the otologic field using nanoparticles within the past 5 years. In theory, nanoparticle-based drug delivery can enhance the efficiency of delivery to the inner ear and release drugs in a sustained manner [5]. Nanoparticles also provide physical protection* in vivo* for delicate drug structures [6].

## 2. Challenges with Inner Ear Drug Delivery

The first challenge is ensuring the safety of the drug carrier in the middle or inner ear [7]. Nanoparticles have been used extensively in cancer therapy where cell viability is not an important issue; however, safety is an absolute requirement for nanoparticle applications for the treatment of deafness. To increase permeability into cells or to perform gene transfer, a positively charged moiety such as a cell-penetrating peptide can be attached to the nanoparticle [8, 9]. However, because positively charged nanoparticles can be ototoxic through limited biodegradability of the particle, the production of intracellular reactive oxygen species, and damage of cell membranes, their application to hearing loss is limited [10]. In addition, it must be determined whether the constituent materials of the nanoparticles will accumulate in the inner ear and be cleared or remain in the inner ear and whether these materials are toxic to hair cells.

When a drug is administered to the middle ear through intratympanic drug injection, it must pass the round window membrane (RWM) or the annular ligament of the oval window (OW) to reach the inner ear. It remains unclear whether these two windows are crossed through diffusion or endocytosis [11]. However, the drug must remain in the middle ear cavity for a sufficient amount of time and remain in contact with these two windows to be delivered to the inner ear. Unfortunately, drugs that enter the middle ear cavity do not remain and are quickly discharged to the Eustachian tube through mucociliary flow of the middle ear [12]. In response to this, gels have been studied in many animal studies. Other studies have used thermosensitive gels, which exist in a liquid state at room temperature and a gel state at body temperature [13].

Another challenge with intratympanic drug delivery is the low permeability of the RWM and annular ligament of the OW. Although the dominant entry route for the inner ear remains unknown, the RWM seems to be the dominant route. Salt et al. [14] reported distribution of an ionic marker, trimethylphenylammonium (TMPA), in the cochlea after intracochlear injection or application to the round window niche based on direct monitoring using a TMPA selective electrode or sequential collection of perilymph. A total of 65% of TMPA entered through the RWM while 35% entered the vestibule in the vicinity of the stapes. However, in clinical situations, RWM may be blocked by fat or fiber tissue; fat or fiber tissue can interfere with drug delivery through the RWM [15]. Factors such as size, configuration, concentration, liposolubility, electrical charge, and membrane thickness influence permeability [16]. Smaller agents are transported more readily through the RWM. Zou et al. [17] explored size-dependent nanoparticle transport. Three sizes of liposome nanoparticles (95, 130, and 240 nm) were manufactured, and their distribution was measured after transtympanic injection in rats. The 95 nm particles were transported most easily whereas the 240 nm particles were transported least easily. With regard to charge, in rodents cationic ferritin readily passes through the normal RWM, whereas anionic ferritin does not [16, 18].

## 3. Studies Investigating the Uptake or Toxicity of Nanoparticles in the Inner Ear

Wen et al. [19] explored several surface-modified PLGA nanoparticles for inner ear drug delivery, of which poloxamer 407-PLGA nanoparticles showed the greatest cellular uptake and strongest fluorescence based on cochlear imaging. It is possible to analyze quantitatively the amount of nanoparticle entering the cochlea using a near-infrared fluorescence imaging system after cochlea harvest (Table 1). However, a more physiological and accurate method would be to analyze quantitatively the cochlea in a live state. This may be accomplished by isolating the perilymph and determining the concentration of the drug using HPLC. However, it is difficult to quantify drug absorbed in cells of the inner ear (not the perilymph) using this method.

In addition, inner ear drug delivery studies using superparamagnetic nanoparticles and chitosan hydrogel-based nanoparticles have recently been published, and both showed good safety and drug delivery efficiency [20, 21] (Table 1).

## 4. Studies Attempting to Deliver Actual Drugs to the Inner Ear Using Nanoparticles

In recent years, rather than simply investigating the permeation of nanoparticles into the inner ear, a growing number of reports have loaded a drug onto the nanoparticle and transferred it to the inner ear to observe functional changes (Table 2).

Drug delivery using polyethylene glycol-coated polylactic acid (PEG-PLA) nanoparticles has been attempted twice by the same group [22, 23]. This group used cisplatin to deafen guinea pigs after pretreatment systemically or intratympanically with dexamethasone-loaded nanoparticles. In both studies, administration of dexamethasone-loaded nanoparticles protected hearing in the 4 kHz and 8 kHz frequencies. In another study in which 6*α*-methylprednisolone was loaded onto nanoparticles using alpha-tocopherol derivatives, cisplatin-induced hearing loss was protected at 10, 14, and 16 kHz [24].

Other reports (excluding the aforementioned studies) have not evaluated changes in hearing after the administration of nanoparticles but instead have analyzed quantitatively the concentration of drug delivered to the inner ear based on high-performance liquid chromatography (HPLC) or fluorescence spectrophotometry [25–28]. One interesting study proposed intratympanic drug injection as a potential brain drug delivery route by analyzing the drug concentration in brain tissue and cerebrospinal fluid (CSF) after intratympanic drug administration [26]. Multiple agent-loaded nanoparticles following intratympanic injection in guinea pigs significantly improved drug distribution within the inner ear, CSF, and brain tissues and protected the brain from cerebral ischemia reperfusion injury.

## 5. Studies Attempting to Deliver Growth Factors to the Inner Ear Using Nanoparticles

The delivery of macromolecules including growth factors to the inner ear may be more clinically useful than simple drug delivery. This is because in individuals with chronic hearing loss, hair cells cannot be regenerated through drug delivery, although they may be facilitated by the delivery of growth factors or genes. Brain-derived neurotrophic factor (BDNF) has been investigated in many animal studies and can preserve the population of spiral ganglion neurons after hair cell loss [37, 38]. It is also considered a candidate growth factor for reversing hearing loss [39].

Recently, several studies have explored the ability of BDNF and NGF to deliver growth factors to the inner ear; however, many of these studies used intracochlear delivery [29–32] (Table 3). Because intracochlear delivery requires surgery and can lead to the loss of remnant hearing by opening the cochlea, it can only be attempted in completely deaf patients, such as for cochlear implant surgery. Intratympanic delivery is more useful because of easy clinical access. However, delivering a large macromolecule such as a growth factor remains challenging.

## 6. Studies Attempting to Deliver Genes to the Inner Ear Using Nanoparticles

A safe and useful nonviral gene delivery system has very high clinical value and can be used to deliver genes to patients with congenital or chronic hearing loss. For example, genes such as Atoh1 can potentially regenerate hair cells in patients with chronic hearing loss, such as presbycusis.

Two recent studies have attempted gene transfer using GFP fluorescence as a positive transfer marker [8, 33] (Table 4). However, it remains difficult to analyze quantitatively the amount of gene delivered to the inner ear using this method because of autofluorescence of the inner ear and the vulnerability of fluorescence intensity to the laser of a confocal microscope. In one of these studies, pRK5-Atoh1-EGFP plasmids were transferred and gene transfer was analyzed quantitatively based on RT-PCR and Western blot, which appeared to be a reliable approach [33]. Although this report did not assess structural or functional changes after* atoh1* delivery, these results demonstrate the possibility of the possibility of nonviral gene delivery through nanoparticles.

Several challenges with gene delivery using nanoparticles must be addressed before it can be applied clinically, such as decreasing the particle size while stably integrating the gene into the particle; administering a gene and nanoparticle complex to the body; protecting the gene from degrading enzymes such as endonuclease; and ensuring that the gene enters the cytoplasm, escapes the endosome, and enters the nucleus.

## 7. Inner Ear Drug Delivery Studies with Imaging Modalities

Direct observation of drugs or nanoparticles in the inner ear with micro-CT or MRI can be used for quantitative analysis of the amount or distribution of a drug delivered to the cochlea. Zou et al. [34] recently injected silver nanoparticles (Ag NPs) intratympanically and observed the distribution of Ag NPs in the middle and inner ear using micro-CT, showing a gradient concentration from the middle ear to the inner ear (Table 5).

It is possible to detect the distribution of nanoparticles within the inner ear using MRI if the nanoparticles contain paramagnetic agents such as gadolinium chelate [40]. In a recent study, gadolinium chelate was encapsulated in a liposome nanocarrier and the distribution of nanoparticles in the inner ear after intratympanic injection was observed using MRI [35]. In addition to nanoparticles containing gadolinium, superparamagnetic iron oxide nanoparticles (SPION) or ceric ammonium nitrate oxidant-stabilized gamma-maghemite nanoparticles were identified in the inner ear using MRI after intratympanic or intracochlear administration [36, 41].

## 8. Conclusion

The use of nanoparticles is a promising therapy for inner ear disease. The ideal nanocarrier should be able to permeate the RWM or the annular ligament on the OW, be capable of specific targeting, provide controlled release of the loaded materials, and be safe in the inner ear. Many studies have attempted to deliver drugs, genes, and growth factors to the inner ear* in vivo*, and promising results have been reported. The safe and effective delivery of drugs or genes will be an important advancement for the treatment of many inner ear diseases, including age-related hearing loss, which is currently a refractory disease.

## Figures and Tables

**Table 1:** Studies investigating the uptake or toxicity of nanoparticles in the inner ear.

Nanoparticle	Size of nanoparticle	Animal	Administration route	Loaded drug or gene	Evaluation time	Evaluation of nanoparticle uptake
Poloxamer 407-PLGA NP [19]	181.5 nm	Guinea pigs	Intratympanic	DiR	At 24 h	Near-infrared fluorescence imaging system, confocal microscope

Superparamagnetic NP [20]	100, 200, and 500 nm (three kinds)	Guinea pigs	Intracochlear	None	At 7 days	Toxicity evaluation by ABR

Chitosan-hydrogel-based NP [21]	160 nm	C57BL/6J mice	Intratympanic	Fluorescent dye	At 24 h	Fluorescent microscopy

PLGA: poly(lactic-co-glycolic acid); NP: nanoparticle; DiR: 1,1′-dioctadecyl-3,3,3′,3-tetramethylindotricarbocyanine iodide; ABR: auditory brainstem response.

**Table 2:** Studies attempting to deliver actual drugs to the inner ear using nanoparticles.

Nanoparticle	Size of nanoparticle	Animal	Administration route	Loaded drug or gene	Evaluation time	Evaluation of nanoparticle uptake
PEG-PLA NP [22]	Not described	Guinea pig, deafened with cisplatin	Intraperitoneal	Dexamethasone	At 3 days	ABR and morphology

PEG-PLA NP [23]	130 ± 4.78 nm	Guinea pig, deafened with cisplatin	Intratympanic	Dexamethasone	At 3 days	ABR and morphology

Multimicellar NP [24]	120.8~159.9 nm	Wistar rats, deafened with cisplatin	Intratympanic	6*α*-methylprednisolone	At 3 days	ASSR

PLGA NP [25]	135 nm with a PDI of 0.17	Guinea pigs	Intratympanic	Salvianolic acid B, tanshinone IIA, and total panax notoginsenoside	At several predetermined time points within 96 h	HPLC of perilymph

PLGA NP [26]	154 nm with PDI 0.007	Guinea pigs	Intratympanic	Salvianolic acid B, tanshinone IIA, and total panax notoginsenoside	At several predetermined time points within 36 h	HPLC of blood, perilymph, CSF, and brain tissue

PLGA-magnetite-NP [27]	482.8 ± 158 nm	Guinea pigs	Intratympanic	Dexamethasone acetate	At 30 min	HPLC of perilymph, RWM, and inner ear tissue

Cubic liquid crystalline NP [28]	138.6~210.9 nm	Guinea pigs	Intratympanic	Earthworm fibrinolytic enzyme	At several predetermined time points within 24 h	Fluorescence microscope and spectrophotometer

NP: nanoparticle; ABR: auditory brainstem response; PEG-PLA: polyethylene glycol-coated polylactic acid; ASSR: auditory steady-state responses; RWM: round window membrane.

**Table 3:** Studies attempting to deliver growth factors to the inner ear using nanoparticles.

Nanoparticle	Size of nanoparticle	Animal	Administration route	Loaded drug or gene	Evaluation time	Evaluation of nanoparticle uptake
Silica supraparticle [29, 30]	500 *μ*m (porous structure)	Deafened guinea pigs	Intracochlear	BDNF	At 4 weeks	Survival of SGNs

Phytantriol lipid-based crystalline NP [31]	215.6~227.2 nm	Guinea pigs, deafened with cisplatin	Intratympanic	NGF	At several predetermined time points within 24 h	ELISA assay of cochlear fluid

Nanoporous PGA NP [32]	1.8–3.2 *μ*m	Guinea pig, deafened with aminoglycoside	Intracochlear	BDNF	At 20 days	Morphology

BDNF: brain-derived neurotrophic factor; SGN: spiral ganglion neurons; NP: nanoparticle; NGF: nerve growth factor; PGA: poly(L-glutamic acid).

**Table 4:** Studies attempting to deliver genes to the inner ear using nanoparticles.

Nanoparticle	Size of nanoparticle	Animal	Administration route	Loaded drug or gene	Evaluation time	Evaluation of nanoparticle uptake
PHEA NP [16]	103.1 nm	C57/BL6 mice	Intratympanic	GFP plasmid DNA and fluorescent dye	At 48 h	Confocal microscope

Dendrimer-based NP [33]	132 ± 20 nm	Sprague Dawley rats	Intratympanic	Atoh1-EGFP plasmid	At 7 days	Confocal microscope, RT-PCR, and Western blot

NP: nanoparticle; PHEA: poly(2-hydroxyethyl L-aspartamide).

**Table 5:** Inner ear drug delivery studies with imaging modalities.

Nanoparticle	Size of nanoparticle	Animal	Administration route	Loaded drug or gene	Evaluation time	Evaluation of nanoparticle uptake
Silver NP [34]	21 ± 8 nm	Sprague Dawley rats	Intratympanic	None	At 4, 7, and 24 h and at 7 days	Micro-CT

Liposome nanocarrier [35]	115 ± 10 nm	Sprague Dawley rats	Intratympanic	Gd-DOTA	At several predetermined time points within 7 days	MRI, ABR, and inflammatory biological markers

Ceric ammonium nitrate oxidant-stabilized gamma-maghemite NP [36]	50–60 nm	Sprague Dawley rats	Intratympanic	None	At several predetermined time points within 14 days	MRI

NP: nanoparticle; Gd-DOTA: gadolinium-tetra-azacyclo-dodecane-tetra-acetic acid; ABR: auditory brainstem response.
